# Seasonal Differences in the UVA/UVB Ratio of Natural Sunlight Influence the Efficiency of the Photoisomerization of (6‐4) Photoproducts into their Dewar Valence Isomers

**DOI:** 10.1111/php.13361

**Published:** 2020-12-22

**Authors:** Kazuki Nishimura, Hironobu Ikehata, Thierry Douki, Jean Cadet, Shigeki Sugiura, Toshio Mori

**Affiliations:** ^1^ Medical Genetics Research Center Nara Medical University Kashihara Japan; ^2^ Radioisotope Research Center Nara Medical University Kashihara Japan; ^3^ Department of Medical Biochemistry Tohoku University Graduate School of Medicine Sendai Japan; ^4^ Université Grenoble Alpes CEA CNRS IRIG SyMMES Grenoble France; ^5^ University of Sherbrooke Sherbrooke QC Canada; ^6^ Department of Radiation Oncology Nara Medical University Kashihara Japan

## Abstract

The UVA and UVB components of sunlight can produce three classes of bipyrimidine DNA photolesions [cyclobutane pyrimidine dimers (CPDs), pyrimidine (6‐4) pyrimidone photoproducts (6‐4PPs) and related Dewar valence isomers (DewarPPs)]. The UVA/UVB ratio of sunlight is high in winter and low in summer in the Northern Hemisphere. Since UVB radiation produces 6‐4PPs and UVA radiation converts them into DewarPPs through photoisomerization, it is expected that there may be differences in the photoisomerization of 6‐4PPs between summer and winter, although that has never been documented. To determine that, isolated DNA was exposed to natural sunlight for 8 h in late summer and in winter, and absolute levels of the three classes of photolesions were quantified using calibrated ELISAs. It was found that sunlight produces CPDs and 6‐4PPs in DNA at a ratio of about 9:1 and converts approximately 80% of 6‐4PPs into DewarPPs within 3 h. Moreover, photoisomerization is more efficient in winter than in late summer after sunlight irradiation for the same duration, at similar solar UV doses and with the same induction level of CPDs. These results demonstrate that seasonal differences in the UVA/UVB ratio influence the efficiency of the photoisomerization of 6‐4PPs into DewarPPs.

## Graphical abstract

The UVA and UVB components of sunlight produce three main classes of bipyrimidine DNA photolesions. The UVA/UVB ratio of sunlight is high in winter and low in summer in the Northern Hemisphere. Since UVB produces (6‐4) photoproducts (6‐4PPs) and UVA converts them into Dewar valence isomers (DewarPPs) through photoisomerization, it is expected that there may be seasonal differences in the photoisomerization of 6‐4PPs. Indeed, photoisomerization is more efficient in winter than in late summer after sunlight irradiation, demonstrating that seasonal differences in the UVA/UVB ratio influence the efficiency of the photoisomerization of 6‐4PPs into DewarPPs.

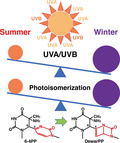

## INTRODUCTION

**Figure 1:**
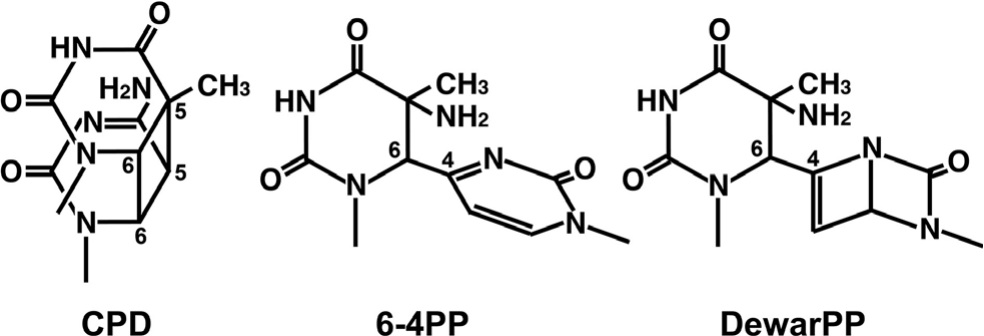
Schematics showing the three main classes of solar UV‐induced DNA damage. The structures represent photolesions formed at a TC sequence. Similar photolesions are produced at TT, CT and CC sequences.

There is overwhelming evidence showing that increased exposure to the UV component of natural sunlight is the major cause of human skin cancers (1, 2). It is accepted that the UV induction of DNA damage is the main initiating event in solar carcinogenesis. Analysis of DNA isolated from skin tumors reveals that the majority of mutations occur at dipyrimidine sites, especially at TC and CC sequences (3, 4), which suggests the major importance of pyrimidine dimer‐type damage among the various types of photolesions. This is supported by the evidence that patients with xeroderma pigmentosum, a genetic disorder involving in most cases defective repair of these pyrimidine dimers, have an extremely high incidence of skin cancers on sunlight‐exposed areas (5). The UV spectrum is subdivided into three regions: UVC (100–280 nm), UVB (280–320 nm) and UVA (320–400 nm). Since wavelengths lower than 290 nm are absorbed by stratospheric ozone, solar UV photons reaching the Earth’s surface is a combination of UVB (290–320 nm) and UVA (320–400 nm). Natural sunlight efficiently induces three classes of pyrimidine dimers including cyclobutane pyrimidine dimers (CPDs), pyrimidine (6‐4) pyrimidone photoproducts (6‐4PPs) and their Dewar valence isomers (DewarPPs) in cellular DNA (6, 7) (Fig. 1).

These photolesions occur because UVB is directly absorbed by cellular DNA and produces CPDs and, to a lesser extent, 6‐4PPs (8). UVA also induces CPDs through direct excitation with however a much lower efficiency (9, 10, 11). Simultaneously, initially UVB‐produced 6‐4PPs are converted into DewarPPs through photoisomerization by the UVA photons of solar radiation (11, 12, 13). This indicates that natural sunlight that includes both UVB and UVA is an appropriate UV source to investigate the induction of each class of pyrimidine dimer in DNA, because firstly, their distribution, particularly 6‐4PPs and DewarPPs, may vary greatly depending on the duration of sunlight exposure. Indeed, a pioneering work based on immunological measurements reported the efficient conversion of 6‐4PPs into DewarPPs following exposure to natural sunlight (6). However, since measurements of these photoproducts have been performed in a relative way, they need to be confirmed using a quantitative method, which is essential for comparison between different types of photolesions. Secondly, the intensity of natural sunlight is impacted by geo‐orbital (latitude, season, time of day, etc.) and environmental (clouds, ozone layer, pollutants, etc.) factors. Indeed, the formation and distribution of DNA damage including CPDs and 6‐4PPs by natural sunlight at different time of day, at different latitudes and at different ozone layer thickness has been investigated (14, 15, 16, 17). It is known that the seasonal variation of the “effective” ozone layer results in an inverse correlation with the seasonal variation of UVB intensity (18). Consequently, there is a significant difference in the UVA/UVB ratio between summer (including July, August and September) with low values (20.4–23.2) and winter (including January and December) with high values (53.5–56.5) in central Japan (19, 20) (values in parentheses are cited from Ref. (19)). Considering that UVB produces 6‐4PPs and that UVA converts them into DewarPPs by photoisomerization, it is reasonable to expect that there may be differences in the photoisomerization of 6‐4PPs into DewarPPs between summer and winter. However, such a study has never been done.

To test that hypothesis, isolated DNA was exposed to natural sunlight as long as 8 h over four clear days in late summer (September) and in winter (December). Exposure to sunlight was performed between 11 a.m. and 1 p.m. in order to average variations of hourly UV doses and daily differences in the UVA/UVB ratio (21). To emphasize that September is categorized as a summer month including July and August with a low UVA/UVB ratio, it is denoted as “late summer.” One type of immunological assay, an enzyme‐linked immunosorbent assay (ELISA), is useful for the measurement of pyrimidine dimers because of its simplicity, sensitivity and reproducibility (22), but it provides only relative yields of formation. To determine absolute levels of pyrimidine dimers, we used a quantitative approach (11, 23) by calibrating conventional ELISAs through the determination of photolesion levels in an UVB‐irradiated DNA sample by high‐performance liquid chromatography tandem mass spectrometry (HPLC‐MS/MS). HPLC‐MS/MS is a highly sensitive and accurate technique that can quantify absolute frequencies of CPDs, 6‐4PPs and DewarPPs at TT, TC, CT and CC sequences (8, 24). Although we have already utilized an UVB‐exposed DNA for the calibration of CPDs and 6‐4PPs (23), we prepared a UVB + UVA‐exposed DNA sample for the calibration of DewarPPs by further irradiation of initially UVB‐exposed DNA with 90 kJ m^−2^ UVA. Using calibrated ELISAs with the two types of calibration DNAs, it was possible to measure the absolute levels of all three classes of pyrimidine dimers. Thus, these measurements have allowed the determination of the efficiency of the photoisomerization of 6‐4PPs into DewarPPs in late summer and winter, respectively.

## MATERIALS AND METHODS

### UV‐exposed calibration DNAs

**Figure 2:**
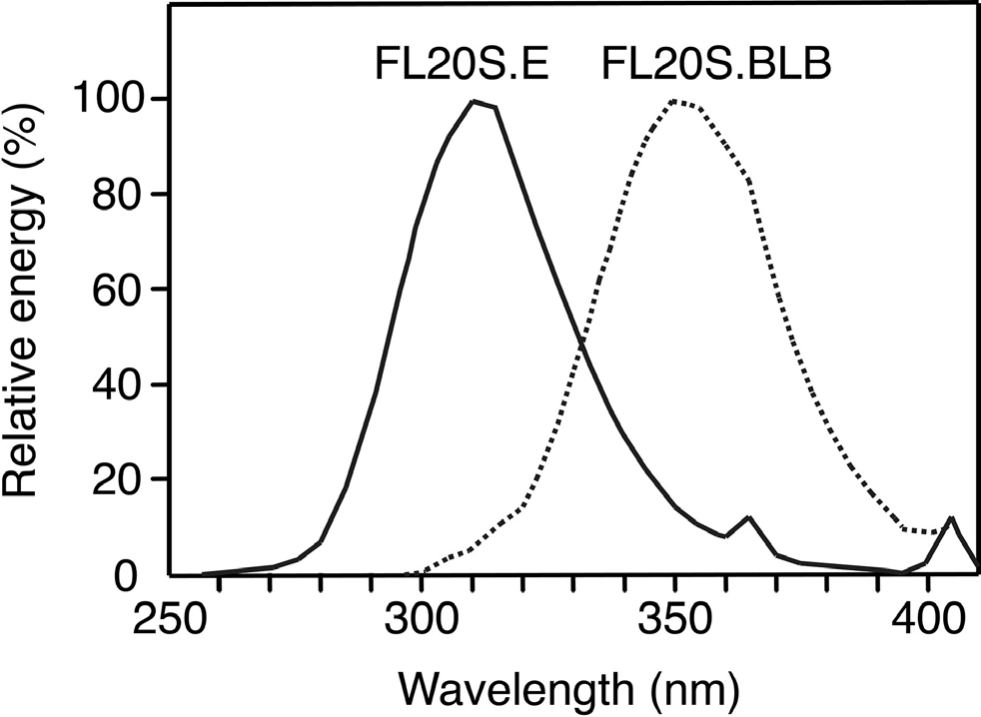
Relative spectral energy outputs of the UVA and UVB lamps. Data for UVB (FL20S.E, solid line) and UVA (FL20S.BLB, dotted line) were supplied by the manufacturer.

Calf thymus DNA (D1501, Sigma‐Aldrich) was dissolved in water at a concentration of 450 µg mL^−1^ in 60 mm dishes and was then irradiated with 50, 100, 200, 300, 400 and 600 J m^−2^ UVB (broadband UVB with a 313 nm peak; FL20S.E lamp, Toshiba, Japan) which was filtered through a Kodacel TA407 sheet to exclude wavelengths below 275 nm. We call this DNA the “UVB‐exposed calibration DNA.” Part of the UVB‐exposed calibration DNA samples prepared by irradiation at doses of 200, 400 and 600 J m^−2^ (400 µg mL^−1^) in 60 mm dishes on ice were further exposed to 90 kJ m^−2^ UVA (broadband UVA with a 350 nm peak; FL20S.BLB lamp, Toshiba) (Fig. 2). We call this DNA the “UVB + UVA‐exposed calibration DNA.”

UVB and UVA dosimetry was performed using a UV radiometer equipped with UVR‐30 and UVR‐36 sensors, respectively (Topcon, Tokyo). Levels of the three classes of photolesions (CPDs, 6‐4PPs and DewarPPs) formed in the UVB‐exposed calibration DNA and in the UVB + UVA‐exposed calibration DNA were quantified by HPLC‐MS/MS as previously described (8, 24).

### Natural sunlight irradiation

**Figure 3:**
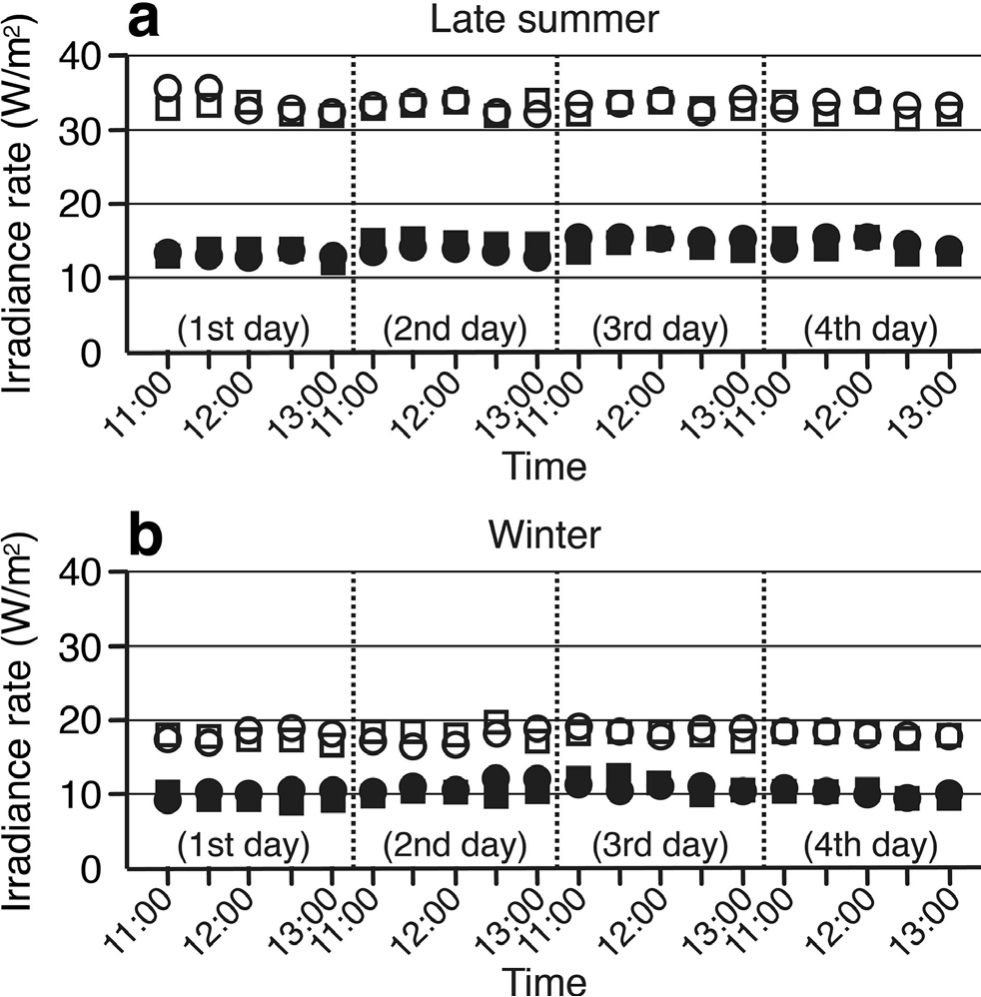
Intensity variations of the sunlight during exposure to DNA samples. (a) The solar UV intensities were monitored as the irradiance rate along with the exposure to DNA samples over four clear days in late summer (Exp. 1, circles; Exp. 2, squares) using a UV radiometer equipped with UVB (UVR‐30, closed symbols) and UVA (UVR‐36, open symbols) sensors. (b) Similar monitoring was performed in winter (Exp. 3, circles; Exp. 4, squares) using the same UV radiometer. It should be noted that the readings with those sensors were just used for a relative indicator of the solar UV intensity and did not reflect real values of the irradiance rates of the UVA and UVB components of sunlight.

Calf thymus DNA was dissolved in water at 400 µg mL^−1^ in 60 mm dishes. DNA samples maintained on ice were exposed to natural sunlight for a maximum of 2 h per day between 11 a.m. and 1 p.m. They were exposed for 1, 2, 3, 4, 5, 6, 7 and 8 h and for each hr during the 8 h exposure over a maximum of four clear days. Sunlight irradiation was performed in late summer (Exp. 1; September 5 and 16–18, 2018; Exp. 2; September 19, 22, 23 and 28, 2018) and in winter (Exp. 3; December 10 and 13–15, 2019; Exp. 4; December 15, 16, 24 and 25, 2019) at the Nara Medical University campus (Kashihara, Japan: 34.5^o^N–135.8^o^E). DNA samples were kept at 4 ^o^C during interruption of sunlight exposure and were frozen at −80 °C after completion of solar irradiation. The typical spectral distribution of sunlight energy in central Japan was reported in a previous study (25). Variations of the solar UV intensity were monitored using a UV radiometer equipped with UVR‐30 and UVR‐36 sensors (Fig. 3).

### Enzyme‐linked immunosorbent assays (ELISAs)

Detection of CPDs, 6‐4PPs and DewarPPs in sunlight‐exposed DNA samples was achieved by ELISA using TDM‐2, 64M‐2 and DEM‐1 monoclonal antibodies, respectively (26, 27), with some modifications. Briefly, 96‐well polystyrene flat‐bottom microplates (Thermo Scientific, nontreated, clear, Cat. No. 260895), precoated with 0.0001% protamine sulfate, were coated in quadruplicate with heat‐denatured sample DNA (10 ng/well for CPDs, 200 ng/well for 6‐4PPs, 500 ng/well for DewarPPs). After blocking with 2% fetal bovine serum, each class of dimeric photolesions was detected with TDM‐2 (1/1000), 64M‐2 (1/1000) or DEM‐1 (1/10 000), followed by goat antimouse IgG (H + L) conjugated to biotin (1/2000; Fitzgerald, Acton, MA, 43R‐1334) and then streptavidin‐peroxidase (1/10 000; Thermo Fisher, Cat. No. 43‐4323). After treatment with *o*‐phenylenediamine (OPD) and H_2_O_2_, the absorbance of colored products derived from OPD was measured at 492 nm.

### Quantification of photolesion levels by calibrated ELISAs

In each ELISA, photolesions in sunlight‐exposed DNA samples were measured along with those in the UV‐exposed calibration DNA in the same plate. Based on the relationship between the antibody binding values and their photolesion levels in the UV‐exposed calibration DNAs, the levels of photolesions in sunlight‐exposed DNA samples were calculated. The UVB‐exposed DNA was used for calibration of CPDs and 6‐4PPs, and the UVB + UVA‐exposed DNA was used for calibration of DewarPPs.

## RESULTS AND DISCUSSION

### Utilization of calibrated ELISAs for the absolute quantification of dimeric DNA lesions

**Figure 4:**
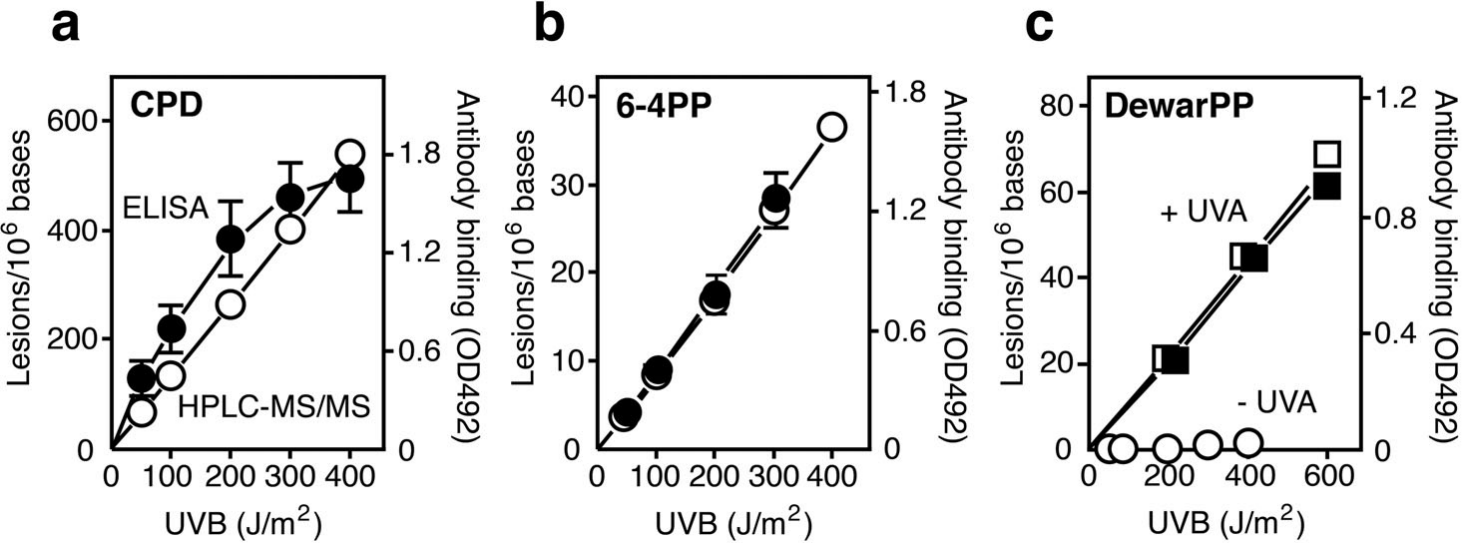
Relationships between levels of photolesions and their antibody binding values in UV‐exposed calibration DNAs. Levels of CPDs (a) and 6‐4PPs (b) in UVB‐exposed calibration DNA were quantified using HPLC‐MS/MS (open symbols). Levels of DewarPPs (c) in the UVB + UVA (90 kJ m^−2^)‐exposed calibration DNA and in the UVB‐exposed calibration DNA were quantified using HPLC‐MS/MS. CPDs (a) and 6‐4PPs (b) in the UVB‐exposed calibration DNA, and DewarPPs (c) in the UVB + UVA‐exposed calibration DNA were detected by ELISA (closed symbols). Each point represents the mean (± SD) of two (HPLC‐MS/MS) or three (ELISA) experiments.

Although ELISAs to detect each of the three classes of pyrimidine dimers are widely used because of their simplicity, sensitivity and reproducibility (22), they provide only a relative estimate that cannot be used for comparison purpose. In this study, we calibrated ELISAs to quantify the absolute levels of photolesions using the UVB + UVA‐exposed calibration DNA as well as the UVB‐exposed calibration DNA in conventional ELISAs. Thus, we quantified the levels of photolesions in the two types of calibration DNAs using HPLC‐MS/MS. The levels of CPDs, 6‐4PPs and DewarPPs for each type of dipyrimidine (TT, TC, CT and CC) sites were determined, summed for each photolesion and plotted (Fig. 4).

HPLC‐MS/MS analysis revealed that the levels of CPDs and 6‐4PPs increased linearly as a function of the dose in the UVB‐exposed DNA. The levels of DewarPPs also increased linearly with increasing doses in the UVB + UVA‐exposed DNA, but no significant level of DewarPPs was produced in the UVB‐exposed DNA, confirming previously reported results (9). The yields of CPDs, 6‐4PPs and DewarPPs in the UV‐exposed calibration DNAs were calculated as 1.31, 0.093 and 0.11 lesions/10^6^ bases per J m^−2^, respectively. This indicates that the relative formation of CPDs and 6‐4PPs was 93:7. Higher yields of DewarPPs in the UVB + UVA‐exposed DNA than those of 6‐4PPs in the UVB‐exposed DNA may reflect that the small UVB component present in the broadband UVA produces significant levels of 6‐4PPs upon exposure to the high dose of 90 kJ m^−2^ which are then converted into DewarPPs by UVA. In parallel, the CPDs and 6‐4PPs in the UVB‐exposed calibration DNA, and DewarPPs in the UVB + UVA‐exposed calibration DNA were detected by ELISA. Antibody binding to 6‐4PPs and to DewarPPs increased linearly with increasing doses in calibration DNAs, but antibody binding to CPDs was dose‐dependent and not linear reaching a plateau at a level of ~ 500 lesions/10^6^ bases. These are consistent with previous ELISA measurements of CPDs and 6‐4PPs (23). These results suggest that high frequencies of CPDs in DNA reduce the efficiency of antibody binding to the target CPD sites because of saturation and therefore lead to a nonlinear dose–response curve. This is not the case for 6‐4PPs and DewarPPs that are generated in lower yields than CPDs. The calibrated ELISAs based on the three dedicated monoclonal antibodies and the two types of calibration DNAs enabled us to quantify the absolute levels of the three classes of photolesions in sunlight‐exposed DNA.

### Quantification of the levels of three classes of dimeric photolesions in sunlight‐exposed DNA

**Figure 5:**
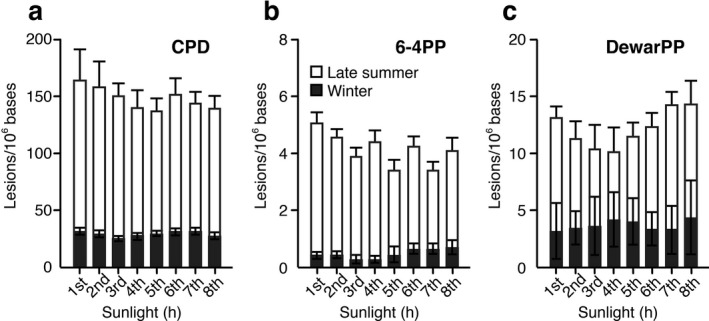
Hourly formation levels of photolesions in DNA samples following 8 h of sunlight exposure. Photolesion levels (a; CPD, b; 6‐4PP, c; DewarPP) were quantified with calibrated ELISAs. Open bars show sunlight exposure in late summer, and closed bars show sunlight exposure in winter. Each bar represents the mean (± SD) of six measurements in two experiments.

DNA samples were exposed to natural sunlight for up to 8 h over four clear days. Sunlight irradiation was limited to a maximum of 2 h per day between 11 a.m. and 1 p.m. in order to average variations of hourly UV doses. The solar UV intensity was monitored using a UV radiometer, but no large variations during exposure were observed (Fig. 3). To check low levels of variation among the individual 1 h solar UV doses during the 8 h exposure, photolesion levels in sunlight‐exposed DNA were quantified every hour using calibrated ELISAs (Fig. 5).

As expected, relatively similar levels of hourly CPD formation were observed among the 8 individual DNA samples exposed in late summer and winter. Late summer sunlight produced average hourly levels of 147.5 ± 10.3 CPDs/10^6^ bases, while winter solar radiation produced those of 29.0 ± 2.4 CPDs/10^6^ bases. Similarly, variations were not large in the levels of hourly formation of 6‐4PPs and DewarPPs among those samples exposed during the two different seasons. These findings indicate that the solar UV intensity is roughly proportional to the duration of sunlight exposure. It is worth mentioning that calibrated ELISAs are sensitive enough to quantify the levels of the three main classes of photolesions in DNA exposed to sunlight for 1 h in summer and winter, respectively.

**Figure 6:**
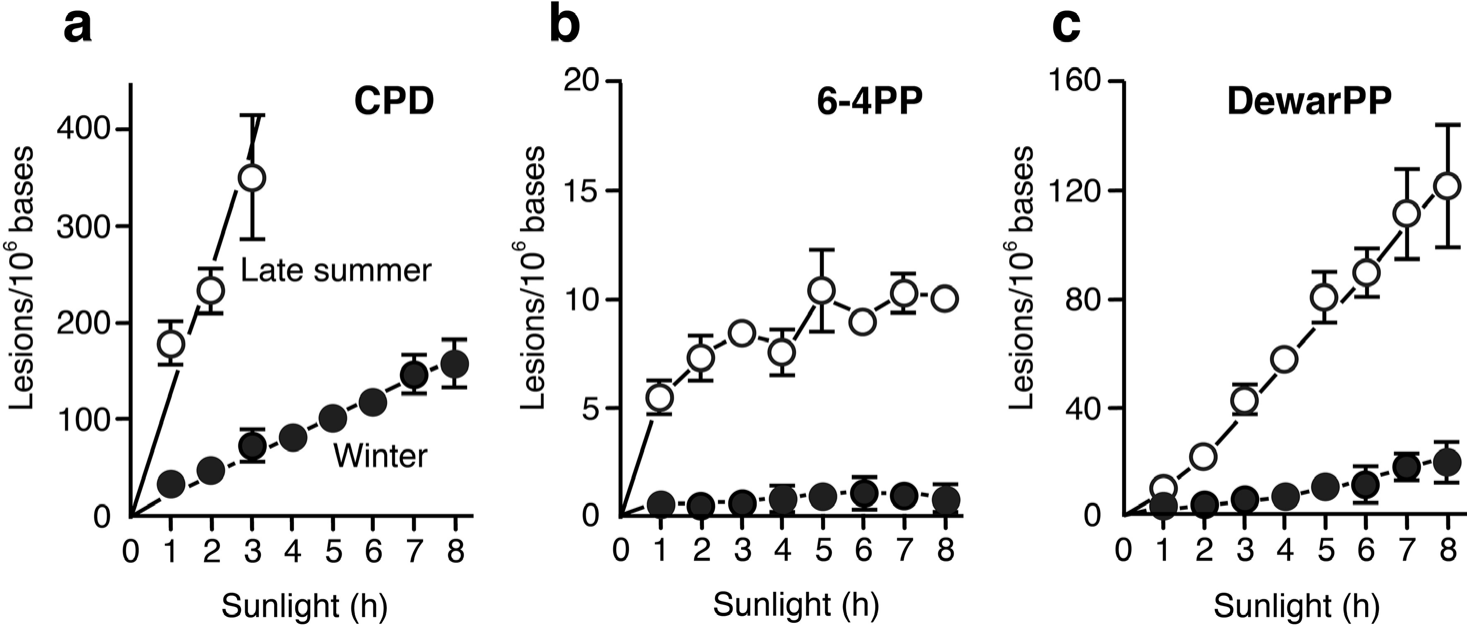
Dose–response curves of the formation of photolesions in DNA samples following exposure to sunlight. Photolesion levels (a; CPD, b; 6‐4PP, c; DewarPP) were quantified with calibrated ELISAs. To quantify high levels of DewarPPs above the calibration, UVC (2 kJ m^−2^)+UVA(90 kJ m^−2^)‐exposed DNA which contained 733 DewarPPs/10^6^ bases was produced. DNA samples which contained 0, 50, 100, 150 and 200 DewarPPs/10^6^ bases were prepared by mixing UVC + UVA‐exposed DNA and nondamaged DNA in different ratios and used for calibrated ELISAs. Open symbols show exposure in late summer, and closed symbols show exposure in winter. Each point represents the mean (± SD) of six measurements in two experiments.

Following the finding that hourly photolesion levels were relatively stable, the dose (duration of sunlight exposure)‐dependent formation of bipyrimidine photoproducts in DNA samples was assessed using calibrated ELISAs (Fig. 6).

The formation of CPDs, 6‐4PPs and DewarPPs was shown to be dose‐dependent but with different patterns. Linear relationship was observed between CPD levels and the duration of sunlight exposure in late summer and winter, confirming the similarity of 1 h UV doses during the exposure. Late summer and winter DNA samples showed yields of 128.7 and 20.4 CPDs/10^6^ bases per 1 h exposure, which corresponded to 97 and 15 J m^−2^ UVB, respectively. In contrast, only a small increase in 6‐4PP levels is noted during the early 3 h sunlight exposure in late summer. However, the formation of 6‐4PPs does not increase significantly with further exposure reaching a plateau at the low level of ~ 10 lesions/10^6^ bases. This indicates that 6‐4PPs induced by 1 h of solar irradiation are almost completely converted into DewarPPs within 3 h of sunlight exposure because of the very efficient photoisomerization by UVA. Indeed, the level of DewarPPs increased dose‐dependently according to a quadratic curve during the early 3 h exposure and a linear curve thereafter. The formation of a quadratic curve is consistent with the fact that the formation of each DewarPP requires two photons, one for the UVB‐induced formation of 6‐4PP, the second for its UVA‐mediated isomerization (13, 28). The linear increase in DewarPP levels after 3 h of sunlight exposure may result from the equilibrium between 6‐4PP formation and its photoisomerization, which causes a constantly low level of 6‐4PPs as the result of its constant conversion. Approximately 80% and 90% of 6‐4PPs were converted into DewarPPs after 3 h and 7 h exposure to sunlight, respectively. Similar dose–response curves were observed in winter DNA samples although levels of 6‐4PPs and of DewarPPs were much lower. Our present findings, on the basis of the absolute quantification of photoproducts, confirmed earlier pioneering work in which it was demonstrated that the majority of 6‐4PPs were converted into DewarPPs in cellular DNA following irradiation with natural sunlight (6). This was also the case in other studies showing that DewarPPs are generated when cells are exposed to a combination of UVB and UVA radiations (9, 11, 29, 30).

Interestingly, as observed for the CPD formation, we noticed linear relationships between the combined levels of 6‐4PPs and DewarPPs and the duration of sunlight exposure in late summer and winter with yields of 16.9 and 2.45 lesions/10^6^ bases per 1 h exposure, respectively. A comparison of these yields revealed that the relative formation of CPDs and the sum of 6‐4PPs and DewarPPs was 88:12 in late summer and 89:11 in winter. A similar ratio was observed for CPDs and the sum of 6‐4PPs and DewarPPs on isolated DNA (85:15) that were exposed to natural sunlight for 12 h on the deck of a boat during a cruise in the Pacific Ocean (7). Similar trend was also noted for bacterial plankton (88:12) and eukaryotic plankton (88:12) on the boat, and in human skin models (90:10) exposed to solar‐simulated radiation (SSR) (31). In isolated DNA, about 90% of 6‐4PPs were converted into DewarPPs, which is consistent with our results. Thus, our findings in agreement with published results indicate that natural sunlight produces CPDs and 6‐4PPs in DNA at a ratio of about 9:1 and converts approximately 80% of 6‐4PPs into DewarPPs within 3 h.

The efficient UVA‐mediated conversion of 6‐4PPs into DewarPPs may have a biological role. It has been reported that the efficiency of repair of DewarPPs is similar to that of 6‐4PPs in human cells (30, 32). However, there may be a difference in the mutagenic potential of these two classes of DNA photolesions. It has been demonstrated by transfecting single‐stranded vectors containing a single photolesion into SOS‐induced *E. coli* cells that TC DewarPPs are more mutagenic than TC 6‐4PPs (6, 33) although TT DewarPPs are less mutagenic than TT 6‐4PPs (34). Considering that SSR produces the sum of TC 6‐4PPs and TC DewarPPs in yields similar to TC CPDs (9) and that TC sequence is one of the mutational hotspots in skin tumors (2, 3), the photoisomerization of TC 6‐4PPs into TC DewarPPs could contribute to increases in mutation induction and of skin cancers by natural sunlight. DewarPPs become the second most frequent type of photoproducts after 1 h of sunlight exposure and may lead to mutations. Thus, DewarPPs are environmentally and biologically relevant photolesions.

### Comparison of the photoisomerization of 6‐4PPs into DewarPPs under sunlight exposure in late summer and winter

**Figure 7:**
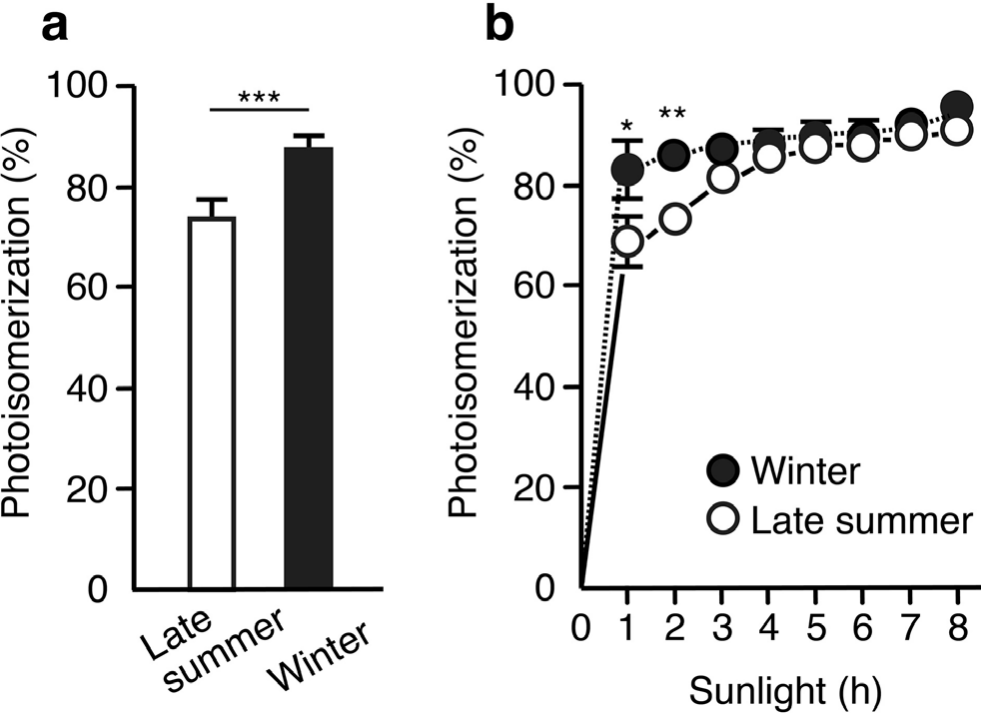
Photoisomerization extent of 6‐4PPs into their DewarPPs in DNA samples exposed to sunlight in late summer and winter. (a) Using the hourly photolesion levels of Fig. 5b,c, 6‐4PP photoisomerization upon 1 h sunlight exposure was calculated. Each bar represents the mean (± SD) of 16 experiments. (b) Using the photolesion levels of Fig. 6b,c, 6‐4PP photoisomerization upon 1–8 h sunlight exposure was calculated. Each point represents the mean (± SD) of six measurements in two experiments. *p* values were calculated using an unpaired two‐tailed Student’s *t* test. *, *P* < 0.05; **, *P* < 0.01; ***, *P* < 0.005.

It is known that there is a seasonal variation in the UVA/UVB ratio of natural sunlight (19, 20). In central Japan, summer (including July, August and September) shows low variations (20.4–23.2) while in winter (including January and December) variations are larger (53.5–56.5), resulting in a significant difference of more than 2.3‐fold. Since UVB produces 6‐4PPs and UVA subsequently converts them into DewarPPs through photoisomerization, it is expected that winter sunlight is more efficient in 6‐4PP photoisomerization than summer solar radiation. To test this hypothesis, we checked for possible differences in photoisomerization efficiency in late summer and winter. Based on the hourly photolesion levels (shown in Fig. 5b,c), the extent of photoisomerization of 6‐4PPs into DewarPPs upon 1 h sunlight exposure in late summer and winter was calculated using the equation [100 x DewarPP / (6‐4PP + DewarPP)] (Fig. 7a).

It was found that the photoisomerization triggered by 1 h sunlight exposure was significantly higher in winter (87.0 ± 2.9) than in late summer (73.6 ± 3.8). Moreover, based on the photolesion levels shown in Fig. 6b,c, the photoisomerization of 6‐4PPs into DewarPPs upon exposure to 1–8 h sunlight was calculated (Fig. 7b). Once again, sunlight in winter showed significantly higher photoisomerization ability than in late summer after 1 h and 2 h exposure. After more than 3 h exposure, photoisomerization efficiency gradually saturated reaching a plateau with a level of ~ 90% in both season samples. These findings demonstrate that photoisomerization of 6‐4PPs is higher in winter than in late summer after solar irradiation for the same duration. However, there is a large difference in UV doses between the 1 h sunlight exposure in late summer and in winter. Since the formation ratios of CPDs (Fig. 6a) and of combined 6‐4PPs and DewarPPs in late summer and in winter are 6.3 (128.7/20.4) and 6.9 (16.9/2.45), respectively, it is suggested that sunlight in late summer contains about a 7‐fold higher UV dose than sunlight in winter. Thus, any difference in photoisomerization efficiency upon exposure to similar solar UV dose in late summer and winter is provided through the comparison of the value (68.9 ± 5.8) calculated upon 1 h exposure in late summer with that (93.2 ± 1.8, saturated) obtained after a 7 h exposure in winter (Fig. 7b). This demonstrates that winter solar radiation induces a much more efficient 6‐4PP photoisomerization than exposure to a similar dose of sunlight in late summer, supporting our hypothesis. Moreover, it is possible to compare the photoisomerization at the same level of CPD formation. Under irradiation conditions that generate ~ 200 CPDs/10^6^ bases, 96.5% (saturated) and 74.2% of the photoisomerization proceeded upon sunlight exposure in winter (UVA/UVB ratio: 54) and late summer (UVA/UVB ratio: 23), confirming the results mentioned above (the values in parentheses are mentioned in Ref. (19)). Interestingly, at the same level of CPD formation, SSR (UVA/UVB ratio: 15) showed only 36.5% of photoisomerization extent in human skin models (31). This supports our hypothesis that the UVA/UVB ratio in sunlight parallels the level of photoisomerization of 6‐4PPs into DewarPPs. Thus, it is evident that seasonal differences in the UVA/UVB ratio influence the efficiency of 6‐4PP photoisomerization, because photoisomerization is more efficient in winter than in late summer after sunlight irradiation for the same duration, at similar solar UV doses, and with the same induction level of CPDs. The UVA/UVB ratio of sunlight depends on latitude, altitude, time of day and other factors, in addition to the season. Therefore, these factors need to be taken into account when designing and analyzing field experiments including the quantification of DNA photolesions.
